# Exploring the Anticancer Potential of *Coriolus versicolor* in Breast Cancer: A Review

**DOI:** 10.3390/cimb47100808

**Published:** 2025-10-01

**Authors:** Marta Ziaja-Sołtys, Magdalena Jaszek

**Affiliations:** 1 Department of Biology with Genetics, Medical University of Lublin, Witolda Chodźki 4A Street, 20-093 Lublin, Poland; 2 Department of Biochemistry, Maria Curie-Skłodowska University, Akademicka Street 19, 20-033 Lublin, Poland

**Keywords:** *Coriolus versicolor*, breast cancer, adjuvant therapy, polysaccharides, extract, mushroom

## Abstract

Breast cancer remains a leading cause of morbidity and mortality among women globally, with increasing incidence projected in the coming years. Despite advances in standard oncologic therapies, there is a growing interest in supportive interventions that enhance treatment efficacy and reduce adverse effects. This review critically evaluates preclinical and clinical data on the medicinal mushroom *Coriolus versicolor* and its bioactive compounds—primarily polysaccharide-K, polysaccharopeptide, and laccase—as potential adjuvants in breast cancer therapy. A systematic PubMed search identified 11 original studies from 2010 to 2025 examining the impact of *C. versicolor* on breast cancer cell lines, animal models, and human subjects. Findings consistently demonstrate antiproliferative, pro-apoptotic, necroptotic, anti-invasive, and immunomodulatory effects across various breast cancer subtypes, including triple-negative breast cancer. One phase I clinical trial also reported good tolerability and immunological benefits in patients post-chemotherapy. The review highlights molecular mechanisms involving apoptosis, necroptosis, and modulation of the tumor microenvironment. While promising, these results underscore the need for standardized preparations, pharmacokinetic data, and larger placebo-controlled trials. Overall, *C. versicolor* shows potential as a safe, natural adjunct to conventional therapy, offering prospects for integrative strategies in breast cancer management.

## 1. Introduction

### 1.1. Breast Cancer: Epidemiology, Clinical Significance, Limitations of Current Therapies

Breast cancer is diagnosed in 2.3 million women worldwide each year. This disease is also the leading cause of death in women. Such high morbidity and mortality are caused, among other things, by the high invasiveness of breast cancer cells leading to metastasis [1].

According to the Global Cancer Observatory: Cancer Tomorrow reports, by 2030, the projected number of new cases of breast cancer worldwide will reach 2.7 million, while the number of deaths will be close to 0.9 million [2,3].

The incidence of breast cancer is expected to increase. Especially in developing countries, due to the current lifestyle of women characterized by late pregnancies, lower willingness of mothers to breastfeed, younger age at first menstruation, lack of physical activity and poor diet [4,5].

Other factors significantly associated with the development of breast cancer are age, family history, obesity, use of oral contraceptives, menopausal status, smoking, alcohol consumption, and genetic factors [6].

Recent developments in molecular biology methods have provided significant progress in cancer diagnostics and therapy. This translates into earlier detection of the disease and increased treatment effectiveness. Cancer cells are characterized by biochemical and genetic mechanisms that are different from normal cells, which affect the course of therapy and can also lead to resistance to anticancer drugs used [7,8]. Knowledge of the pathophysiology and molecular subtypes of breast cancer definitely facilitates understanding of the development of the disease, its course and prediction of the body’s response to treatment. It also allows for the use of personalized therapy, which increases survival rates and improves the quality of life of patients [5,9,10,11].

For prognostic assessment and clinical decision-making, breast cancer has been divided into three major subtypes based on the presence or absence of molecular markers for estrogen receptor (ER), progesterone receptor (PR), and human epidermal growth factor receptor 2 (ERBB2, Erb-B2 Receptor Tyrosine Kinase 2, also known as HER2). Approximately 70% of patients have hormone receptor-positive (HR+) and HER2-negative breast cancer cells; 15–20% have HER2-positive breast cancer; and 15% have triple-negative breast cancer (TNBC), tumors that lack all three standard molecular markers [5,9,10,11]. Breast cancer survival varies depending on stage, molecular subtype, and histological subtype. In Europe, the 5-year survival rate for women with early breast cancer is 96%, compared with 38% for metastatic breast cancer at diagnosis [12].

The increase in the number of breast cancer survivors is a positive observation, but at the same time, new problems have been noticed related to the side effects of surgical therapy, chemotherapy, radiotherapy, or adjuvant therapy. They have a significant impact on women’s physical, psychosocial, and emotional health [13].

### 1.2. Growing Interest in Supportive Therapies and Natural Products in Oncology

Despite significant progress in early diagnostics, surgery, radiotherapy, chemotherapy, targeted and hormonal therapy of breast cancer, it is still necessary to search for new strategies to support treatment, increase treatment efficacy, reduce its toxicity and improve the quality of life of patients [1].

Therefore, attempts to develop new anticancer drugs using unique metabolites extracted from natural sources, an excellent example of which are fungi, are important.

Currently, over 60% of anticancer drugs used are products based on natural compounds of plant origin, e.g., paclitaxel, podophyllotoxin derivatives and vinca alkaloids (vinblastine and vincristine), while doxorubicin, daunomycin, mitomycin C and bleomycin are examples of anticancer agents obtained from fungi [3,14,15,16,17].

Metabolic pathways related to carcinogenesis are interesting targets for anticancer therapies. It is believed that secondary metabolites derived from edible and medicinal mushroom species can affect many processes related to cancer, which suggests the possibility of their use in combination therapies [17].

In Japan and China, medicinal mushrooms have been approved for use for 30 years as a support for standard treatment of breast cancer and to reduce the side effects of radio- and chemotherapy [16,18,19,20,21].

Therefore, studies on the toxicity and biological effects of dietary supplements of natural origin in cancer in general, and in breast cancer in particular, are of great importance [1].

### 1.3. Coriolus versicolor: Biological and Ethnomedicinal Characteristics

*Coriolus versicolor* (L.) Quèl. (1886) or *Trametes versicolor* (L.) Lloyd (1920) is an arboreal fungus, obligate aerobe, commonly found on logs, stumps, and branches of trees throughout the year, in forested temperate zones of Asia, Europe and North America. It is known as “Yun Zhi” (meaning “cloud-like mushroom”) in China, “Kawaratake” in Japan and Turkey tail in North America because of the characteristic appearance of the cap [22,23,24].

*Coriolus versicolor* (CV) belongs to the class *Agaricomycetes*, in which the so-called alamellae fungi are distinguished—a non-phylogenetic group, including large-fruited forms, with species producing durable, woody fruit bodies [25].

The medicinal value of this mushroom, which has a positive effect on general health, increased vitality and longevity, has been known in traditional Chinese medicine for at least 2000 years [16]. Scientific research suggests that it may also include antimicrobial, antiviral, and anticancer properties [24]. It contains many bioactive substances, the most important of which are polysaccharide Krestin (PSK) and polysaccharide peptide (PSP). These compounds have been approved in Asia for immunotherapy or as stimulants of both non-specific and specific immune responses, as well as for their anti-cancer effects. Numerous in vitro and in vivo studies have shown that these compounds can inhibit the proliferation of cancer cells and support their elimination by the immune system [21,26,27].

In the United States, many naturopaths and integrative oncologists quite commonly prescribe *C. versicolor* products to patients with breast cancer [20]. It is believed that these products, especially in powdered form and taken as a tea, support the body in removing toxins, improve the functioning of the liver, spleen, and immune system [20,23,28,29]. Previous studies on the biological activity of *C. versicolor* have focused mainly on its use in gastrointestinal cancers (stomach cancer, colon cancer) and lung cancers. However, more and more scientific reports indicate its potential usefulness in the context of breast cancer treatment [16,30,31,32].

### 1.4. Chemical Composition of the Coriolus versicolor Mushroom

The results of phytochemical and pharmacological studies show that fungi synthesize primary metabolites, such as polysaccharides and enzymes, and secondary metabolites, e.g., terpenoids, phenolic compounds, which have a wide spectrum of biological activities of therapeutic importance, such as immunostimulatory, antioxidant, antiviral, hypoglycemic, fibrinolytic or thrombolytic [33].

#### 1.4.1. Polysaccharides

The biological anticancer properties of fungi are mainly related to the effect of branched polysaccharides (glucans), glycoproteins or polysaccharides combined with peptides/proteins on the immune response of the organism [26,34,35].

A method of culturing CV mycelia in a bioreactor was developed, and it was shown that they can be isolated from both the mycelia and the post-culture fluid [16]. These are the most thoroughly studied and most biologically active components of this mushroom [5,36]. Commercial products from *C. versicolor* used as adjuvants in cancer therapy are mainly polysaccharide peptides (PSP) from China and polysaccharide krestin (PSK) from Japan, obtained from strains “COV-1” and “CM-101” [16]. These polysaccharides have a molecular weight of about 100 kDa and are chemically similar, differing in the presence of fucose in PSK, while rhamnose and arabinose are present in PSP. The ratio of polysaccharides to peptides is 90–10% in PSP and 60–40% in PSK, respectively. PSK and PSP polysaccharides are water-soluble [24,37,38]. A detailed structural analysis of the chemical composition of *C. versicolor* polysaccharides was described by Awadasseid et al. [39], Zhang et al. [40], Rau et al. [41] and Habtemariam [16] (Table 1).

Chan et al. patented a method for obtaining a highly purified herbal powder extract of *C. versicolor* (Yun Zhi) that inhibits cancer cell proliferation. From another CV preparation, they isolated the active compound ergosta-7,22-dien-3 beta-ol, which also shows anticancer potential (US patents US2007104727A1 and US2009247497A1) [47]. The chemical composition of mushrooms also includes minerals, vitamins (e.g., thiamine, riboflavin, ascorbic acid, and vitamin D), amino acids, and other organic compounds that have beneficial effects on health [26].

#### 1.4.2. Low-Molecular-Weight Secondary Metabolites Isolated from *C. versicolor* and Their Biological Activity

The fruiting body of *C. versicolor* also contains potential pharmacologically active secondary metabolites belonging to low-molecular-weight compounds [16]. Some of them have also shown specific activity against abnormal signaling pathways in cancer cells and have affected cell proliferation and survival, and angiogenesis [19,26,42,43,44].

Terpenes are compounds known for their biological potential, and those isolated from fungi have shown anticancer properties. It has been established that terpenes affect the immune system by regulating the expression of genes encoding proteins involved in the immune response [3]. Wang et al. [46] isolated spiroaxane sesquiterpenes, tramspiroins A–D, lactone 15,16-acetonide and isodrimendiol and funatrol D sesquiterpenes from CV cultures [16,46].

Mushrooms are also a rich source of lectins, carbohydrate-binding proteins that exhibit cytotoxic, antiproliferative, and anticancer properties through various mechanisms of action [3]. Important low-molecular-weight metabolites also include phenolic compounds with antioxidant and redox activity. According to research results, the fruiting bodies of *C. versicolor* of European origin contain numerous compounds of this type belonging to flavonoids (flavones, flavonols, flavanone, flavanols, biflavonoids, isoflavonoids) and hydroxycinnamic acid derivatives [45].

Aqueous extracts of CV also contain significant amounts of baicalin, quercetin, isorhamnetin, catechin, amentoflavone, p-hydroxybenzoic acid, and cyclohexanecarboxylic acid. These compounds are not considered to be major components of the fungus, and further studies are needed to determine their potential contribution to the known biological activities of *C. versicolor* [16,45].

## 2. Materials and Methods

### 2.1. Objective and Rationale of the Article

This review article aims to summarize the current state of knowledge on the therapeutic potential of *C. versicolor* as a supportive agent for the treatment of breast cancer. In particular, the work focuses on the analysis of preclinical and clinical studies on its main bioactive components—polysaccharides PSK (krestin) and PSP (polysaccharidopeptide). Given the growing interest in supportive therapies and the need to improve the efficacy and tolerability of oncological treatment, there is a need to collect and evaluate the available scientific data in this area.

The review includes an analysis of the current literature data from the last 15 years (both preclinical and clinical) on the use of CV for breast cancer therapy. It also describes the identification of molecular and immunological mechanisms by which the components of the mushroom can exert anticancer effects and an assessment of the potential clinical efficacy and safety of preparations containing *C. versicolor* in the context of supporting oncological therapy. Finally, it indicates the current limitations and directions of future research necessary to confirm the efficacy and implementation of these preparations in clinical practice.

The preparation of this review of publications has practical and scientific importance. It allows for the summary of current knowledge and assessment of the level of advancement of research in this field, which can be the basis for the design of future translational studies, randomized trials and possible integration of therapies based on medicinal mushrooms with conventional treatment of breast cancer.

### 2.2. Inclusion and Exclusion Criteria

Inclusion criteria included original research articles published in English in peer-reviewed journals within the last 15 years (i.e., from 2010 to 2025). The search sources included PubMed and studies on the effect of products derived exclusively from the *Coriolus versicolor* fungus on breast cancer cells. Studies using extracts, protein-bound polysaccharides (PBPs), enzymes (e.g., laccase) or whole preparations of *Coriolus versicolor* were considered. Studies on breast cancer cell lines, animal models and clinical trials (if they met the other criteria) were included in the review.

Exclusion criteria included literature reviews, meta-analyses, editorial comments, letters to the editors, and conference papers without complete experimental data. Studies in which *C. versicolor* preparations were used in combination with other fungi or with biological molecules such as monoclonal antibodies, as well as those concerning the use of *C. versicolor* in cancer therapy other than breast cancer, were excluded. Articles published in languages other than English were excluded. Articles published outside the PubMed database were excluded.

### 2.3. PubMed Search Strategy

A literature search was performed in PubMed using the following Boolean query: “*Coriolus versicolor*” [Title/Abstract] and “breast cancer” [Title/Abstract] [48]. The filter was set to include publications from the last 15 years (specifically from 1 January 2010 to 10 May 2025) and the English language. 27 records were identified by searching PubMed. Based on the titles and abstracts, 16 publications that did not meet the inclusion criteria (e.g., reviews, other cancers, other fungi or mixed fungal products) were excluded. Full text analysis was performed on 11 publications. Ultimately, 11 studies that met all the criteria were included in the review.

## 3. Mechanistic Insights into the Antitumor Activity of *Coriolus versicolor*

The anticancer properties of *Coriolus versicolor* (CV) are mediated through multiple molecular pathways. Preclinical data demonstrate that its polysaccharides (PSK and PSP), laccase, and small-molecule metabolites exert effects at both cellular and molecular levels.

The *Coriolus versicolor* fungus, known for its immunomodulatory and cytotoxic properties, is the subject of intensive research on its potential use in breast cancer therapy [24,47].

### 3.1. Cytotoxic and Anti-Proliferative Activity

A review of eleven research articles, including cell and animal models of breast cancer, shows a complex and multifaceted mechanism of action of *C. versicolor* extracts and isolated components such as laccase or protein–polysaccharide complexes. In selected publications, in vitro studies were conducted on the following cell lines: MCF-7 (5 studies), 4T1 (3 studies), HBL-100 (1 study), T47D (1 study), and MDA-MB-231 (2 studies).

Most of the analyzed publications demonstrated significant cytotoxicity of CV preparations against breast cancer cells, measured, among others, by MTT, WST-1 and LDH tests.

Shnyreva et al. (2018) [33] conducted an investigation into the antiproliferative properties of *Coriolus versicolor* extracts, specifically assessing growth inhibition (GI) values—the concentration required to achieve a 50% reduction in cancer cell proliferation. The study demonstrated that a methanol–chloroform (1:1) extract derived from the mycelial biomass of *C. versicolor* (strain It-1), cultivated under solid-state fermentation conditions, exhibited significant inhibitory activity against the HBL-100 human breast tumor cell line (GI_50_ = 6.6 μg/mL) and the T47D breast ductal carcinoma cell line (GI_50_ = 45.0 μg/mL). These findings underscore the potential of *C. versicolor* (strain It-1) as a candidate for anticancer therapy.

Furthermore, aqueous extracts obtained via hot water extraction were evaluated for cytotoxicity. Results indicated no cytotoxic effects toward HBL-100 cells, with an LD_50_ (lethal dose) exceeding 100 μg/mL. The HEK293T human embryonic kidney cell line, used as a control, similarly exhibited an LD_50_ 95 ± 20 μg/mL, suggesting a favorable safety profile for non-cancerous cells [33].

In the study by Yuzugullu Karakus et al. (2025) [49], it was shown that at high concentrations, laccase significantly reduced the viability of hormone-sensitive MCF-7 and hormone-insensitive MDA-MB-231 cells, inducing morphological changes characteristic of apoptosis. Purified TPP-partitioned (three-phase partitioning) laccase, biochemically characterized in detail, was used to study cytotoxicity.

After 24 h of exposure to 5 μM TPP-partitioned laccase, HUVEC (primary human umbilical vein endothelial cells) proliferation remained at ~85%, with only minor reductions observed at 6.18 μM (82.5%) and 6.60 μM (81.2%). In contrast, the same treatment markedly suppressed tumor cell viability, reducing proliferation to 59.8% in MCF-7 and 57.4% in MDA-MB-231 cells. IC_50_ analysis further confirmed greater sensitivity of MCF-7 cells compared to the triple-negative MDA-MB-231 line, with respective IC_50_ values of 6.18 μM and 6.60 μM.

**Figure 1:**
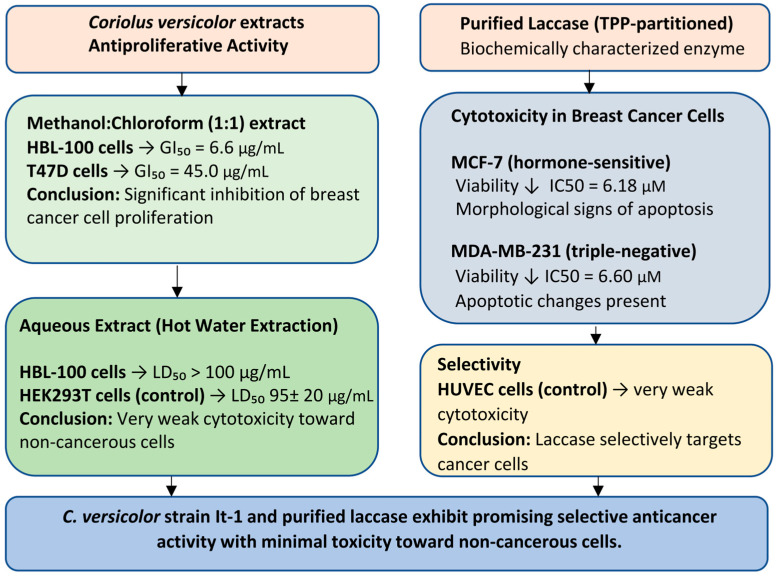
Block diagram summarizing the antiproliferative and cytotoxic effects of *Coriolus versicolor* extracts and purified laccase. The methanol–chloroform extract showed significant growth inhibition in breast cancer cell lines, while aqueous extracts were very weakly toxic to non-cancerous cells. Purified laccase significantly reduced the viability of breast cancer cells and had little effect on healthy endothelial cells, indicating the potential for safe and targeted anticancer therapy.

Similarly, morphological changes indicative of cell death by apoptosis were greater in MCF-7 cancer cells [49] (Figure 1).

### 3.2. Apoptosis Induction and Estrogen Signaling Disruption

The proliferation of hormone-sensitive breast cancer cells is stimulated by estrogens (e.g., 17β-estradiol) through both genomic and non-genomic signaling pathways. These hormones also inhibit apoptosis by modulating the expression of specific apoptotic regulatory proteins, including Bcl-2, p53, and the transcription factor NF-κB. The *BCL2* gene, encoding the anti-apoptotic Bcl-2 protein, is frequently overexpressed in breast cancer cells, a phenomenon attributed to the presence of 17β-estradiol. This overexpression counteracts the release of apoptogenic factors from the mitochondrial intermembrane space, thereby promoting cell survival. Furthermore, breast cancer cells lacking functional p53—a key tumor suppressor protein—exhibit reduced sensitivity to chemotherapy. Similarly, elevated levels of the transcription factor NF-κB are associated with decreased treatment responsiveness. NF-κB regulates critical cellular processes such as apoptosis, cell proliferation, and inflammation, thereby contributing to tumor progression and therapeutic resistance [50,51,52,53].

Chauhan et al. (2019) [54] described an interesting mechanism of the degradation of β-estradiol by laccase from *C. versicolor* immobilized on silver nanoparticles stabilized with Pluronic polymer (AgNPs TrpF1). In the conducted experiment, the anticancer properties of AgNPs TrpF1 were first examined, and for this purpose, the viability of MCF-7 cells was assessed against immobilized laccase, free laccase and AgNPs TrpF1 alone. The results showed that after 48 h, laccase combined with AgNPs TrpF1 at the highest concentration of 120 μg/mL and enzyme activity of 2.0 U/mL almost completely reduced the viability of the tested cells compared to the control sample of free laccase (45 ± 2.0%, 4 mg/mL, 2.0 U/mL) and AgNPs TrpF1 alone, which showed slight anticancer properties. Thus, enzyme immobilization resulted in its greater bioavailability at low concentrations, which translates into greater effectiveness.

Chauhan et al. (2019) [54] described an interesting mechanism of β-estradiol degradation by *C. versicolor* laccase immobilized on Pluronic polymer-stabilized silver nanoparticles (AgNPs TrpF1). In the conducted experiment, the anticancer properties of AgNPs TrpF1 were first investigated by assessing the viability of MCF-7 cells against immobilized laccase, free laccase, and AgNPs TrpF1 separately. The results showed that after 48 h, laccase combined with TrpF1 AgNPs at the highest concentration of 120 μg/mL and enzyme final activity of 2.0 U/mL almost completely reduced the viability of the tested cells (to approximately 5%) compared to free laccase, which reduced the viability to 45 ± 2.0% at a concentration of 4 mg/mL and enzyme final activity of 2.0 U/mL. TrpF1 AgNPs alone exhibited weak anticancer properties (viability decreased to approximately 75%). Therefore, immobilization of the enzyme resulted in its greater bioavailability at low concentrations, which translated into greater efficacy.

The antitumor activity of immobilized laccase was also studied at the molecular level by comparing the mRNA expression of the apoptosis-related genes *BCL-2* (B-cell CLL/lymphoma 2), *p53* (tumor protein 53), and *NF-kβ* (NF-κB (Nuclear Factor kappa B) in MCF-7 cells. A decrease in the levels of *BCL-2* and *NF-kβ* mRNA by 81 and 88%, respectively, and a 3.6-fold increase in the level of *p53* mRNA were observed compared to the control sample. Thus, MCF-7 cells of hormone-dependent cancer in the presence of laccase immobilized on silver nanoparticles stabilized with Pluronic polymer undergo apoptosis due to the reduced availability of 17β-estradiol for ER (estrogen receptors) and the change in the apoptosis signaling pathway PI3K/Akt, (phosphatidyl inositol 3-kinase/v-akt murine thymoma viral oncogene homolog 1 phosphatidylinositol 3-kinase/protein kinase AKT) directly regulated by the level of gene expression for *BCL-2*, *p53* and *NF-kβ* [54].

**Figure 2:**
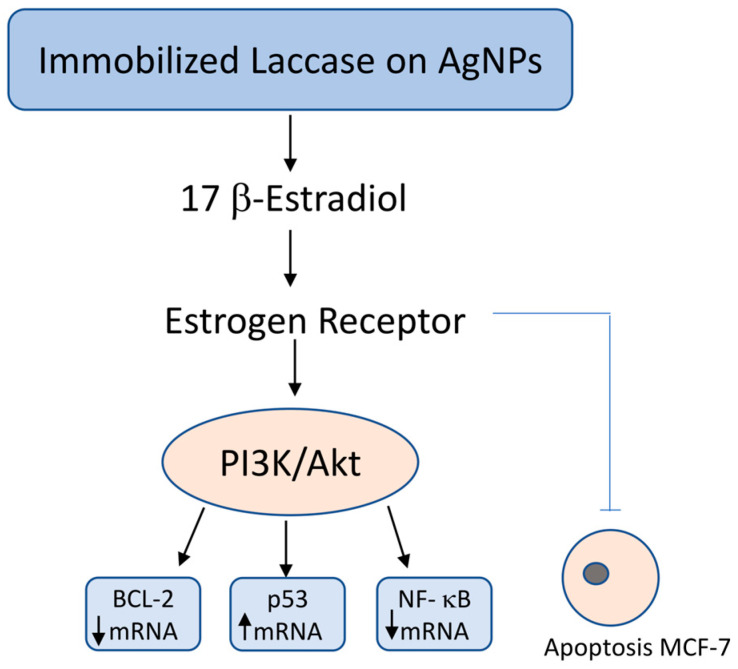
Molecular mechanism of 17β-estradiol degradation by immobilized laccase on silver nanoparticles and its impact on apoptosis in hormone-sensitive MCF-7 breast cancer cells. Immobilized laccase on silver nanoparticles (AgNPs) catalyzes the degradation of 17β-estradiol, reducing its availability for estrogen receptor (ER) activation. This leads to downregulation of the PI3K/Akt signaling pathway, which in turn decreases the expression of anti-apoptotic *BCL-2* and pro-survival *NF-κB*, while simultaneously upregulating the tumor suppressor gene *p53*. The resulting shift in gene expression promotes apoptosis and inhibits proliferation, migration, and invasion of hormone-sensitive MCF-7 breast cancer cells.

*Coriolus versicolor* extracts have been shown to exert anticancer effects in hormone receptor-positive breast cancer cells, such as MCF-7, through multiple mechanisms. Notably, they induce apoptosis via upregulation of the tumor suppressor protein p53 and concomitant downregulation of anti-apoptotic markers BCL-2 and NF-κB. Additionally, interference with estrogen receptor (ER)-mediated signaling has been reported. In particular, laccase enzymes immobilized on nanoparticles derived from *C. versicolor* are capable of degrading 17β-estradiol, thereby disrupting estrogen-driven proliferative pathways in hormone-sensitive breast cancer cells. These findings suggest a dual mechanism involving both pro-apoptotic activity and hormonal modulation, highlighting the therapeutic potential of *C. versicolor* in ER-positive breast cancer management [54] (Figure 2).

### 3.3. Necroptosis Activation

Necroptosis, a form of programmed necrosis, represents a tightly regulated form of cell death distinct from apoptosis.

Necroptosis is a regulated form of cell death (RCD) that is initiated in response to disruptions in cellular homeostasis and is mechanistically distinct from apoptosis. It is driven by the phosphorylation of mixed lineage kinase domain-like protein (MLKL) by receptor-interacting kinases RIPK1 and RIPK3. Activation of this pathway is typically initiated through engagement of cell surface death receptors such as TNFR1 (tumor necrosis factor receptor-1) and FAS, or pattern recognition receptors (PRRs) including Toll-like receptors (TLR3, TLR4) and the cytosolic sensor Z-DNA binding protein 1 (ZBP1).

Central to necroptosis is the activation of RIPK3, which occurs through RHIM (RIP homotypic interaction motif)-mediated interactions. These include RIPK1-RIPK3 binding upon TNFR1 activation, TRIF-mediated recruitment of RIPK3 downstream of TLR3 and TLR4. Once activated, RIPK3 phosphorylates MLKL, inducing its oligomerization and translocation to the plasma membrane [55]. This leads to a series of distinct morphological changes, including cell swelling, increased cellular volume, vacuolization, disruption of the plasma membrane, and the subsequent release of intracellular contents into the extracellular space [56,57,58].

Pawlikowska (2020) [59] and others showed that PBPs isolated from *C. versicolor* at a concentration of 200 mg/mL induce necroptosis in ER-positive MCF-7 cells by activating the RIPK1/RIPK3/MLKL (receptor-interacting protein 1 kinase/receptor-interacting protein kinase 3/mixed lineage kinase domain-like protein) pathway, depending on the TNF-α/TNFR (tumor necrosis factor—α, tumor necrosis factor receptor-1) signaling pathway, but independently of reactive oxygen species (ROS). In a holotomographic microscopy (HT) study, PBPs-stimulated MCF-7 cells showed plasma membrane rupture, a hallmark of programmed cell death via necroptosis. To thoroughly investigate the mechanism of cell death induced by CV PBPs, pharmacological inhibitors of RIPK1 (necrostatin-1, Nec-1), RIPK3 (GSK’872) and MLKL (NSA, necrosulfonamide) kinases, which are antagonists of the necroptotic pathway, were used. These inhibitors were found to protect MCF-7 breast cancer cells from necroptosis and abolish the morphological changes induced by PBPs. It has been established that activation of RIPK1 and engagement of RIPK3 induce necroptosis through a cascade of downstream events, including oligomerization and translocation of MLKL to the plasma membrane, which in turn promotes membrane permeabilization and ultimately results in cell death [59].

**Figure 3:**
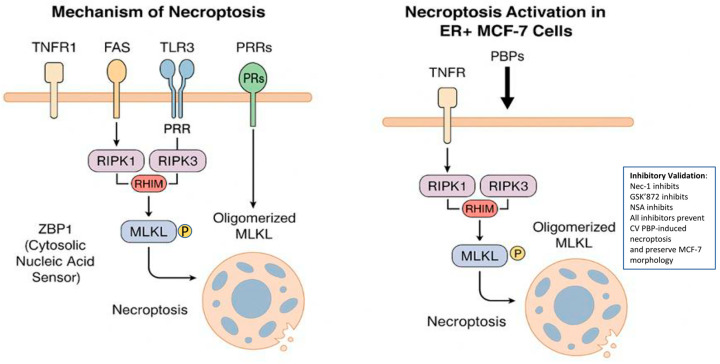
Molecular mechanism of necroptosis and its induction in ER-positive MCF-7 breast cancer cells by protein-bound polysaccharides (PBPs) from *Coriolus versicolor*. Left panel: Schematic representation of the canonical necroptosis signaling pathway. The process is initiated via tumor necrosis factor receptor 1 (TNFR1), FAS, Toll-like receptors (TLR3/4), or cytosolic pattern recognition receptors (ZBP1). Activation of RIPK1 and RIPK3 through RIP homotypic interaction motif (RHIM)-dependent interactions leads to MLKL phosphorylation. Phosphorylated MLKL oligomerizes and translocates to the plasma membrane, culminating in membrane rupture and cell death. Right panel: PBPs from *C. versicolor* trigger necroptosis in estrogen receptor-positive (ER+) MCF-7 cells by stimulating the TNF/TNFR-RIPK1/RIPK3/MLKL axis independently of reactive oxygen species (ROS). Pharmacological inhibition of RIPK1, RIPK3, or MLKL abrogates this process, confirming the involvement of this specific signaling cascade.

Thus, PBPs from CV activate the RIPK1/RIPK3/MLKL axis, triggering necroptotic death in ER+ cells independently of ROS [59]. The induction of necroptosis has emerged as a promising alternative strategy in anticancer therapy. In vitro studies by Pawlikowska et al. [59] have uncovered novel molecular mechanisms through which PBPs exert their effects on cancer cells. Despite the clinical relevance of these compounds, additional in vivo investigations are necessary to validate their therapeutic potential (Figure 3).

### 3.4. Anti-Metastatic Effects

There are numerous tight cellular junctions between breast cancer cells, similar to normal cells. They do not facilitate the invasion process of cancer cells. Therefore, breast cancer cells must lose part of their epithelial phenotype, becoming mesenchymal cells capable of migration and invasion. The next step is the secretion of matrix metalloproteinases (MMPs) by breast cancer cells at the site of the primary tumor, which are essential in the process of cancer cell invasion. Thanks to the activity of these enzymes, cancer cells penetrate the basement membrane into the extracellular matrix and surrounding healthy tissues [60]. The activity of matrix metalloproteinase-9 (MMP-9) is known to be involved in the degradation of the extracellular matrix and basement membrane, and its elevated levels have been associated with cancer progression and reduced patient survival [61].

Luo et al. (2014) [62] studied the antimigratory and antiinvasive potential of polysaccharides from the aqueous extract of *C. versicolor* on mouse breast cancer cell line 4T1, and their antitumor, antimetastatic, and immunomodulatory activity in vivo on a mouse model (female BALB/c, 6–8 weeks old) with 4T1 tumor. These cells are very invasive and form metastases of the primary tumor to the lung, liver, bone and brain within 2 weeks of vaccination, thus this phenomenon corresponds to stage IV breast cancer in humans.

The results of the study demonstrated that the aqueous extract of *Coriolus versicolor* (0.5–2 mg/mL) had no effect on the proliferation of 4T1 cells after 24, 48, and 72 h. However, the CV extract significantly inhibited cell migration (0.5–2 mg/mL after 4–5 h) and invasion (1–2 mg/mL after 9 and 18 h).

**Figure 4:**
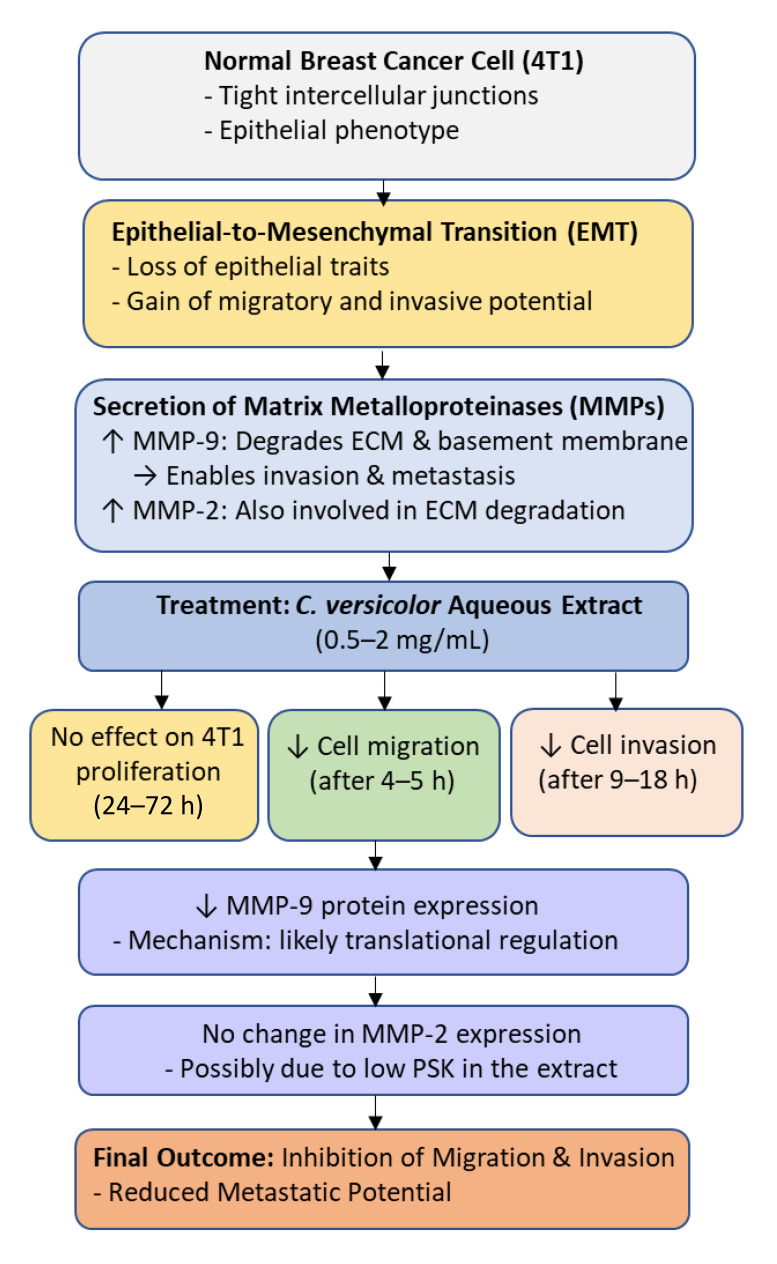
Molecular mechanism underlying the anti-migratory and anti-invasive effects of *Coriolus versicolor* aqueous extract on breast cancer cells (4T1). Treatment with the extract (0.5–2 mg/mL) inhibits epithelial-to-mesenchymal transition (EMT) and reduces cell migration and invasion by downregulating MMP-9 protein expression without affecting MMP-2 or cell proliferation. The inhibition of MMP-9 likely occurs at the translational level, leading to suppression of extracellular matrix (ECM) degradation and reduced metastatic potential.

In vitro studies show that CV prevents the formation of breast cancer metastases. It was found that an aqueous extract of *C. versicolor* significantly reduced the level of MMP-9 protein in 4T1 cells, but did not affect the level of MMP-2 protein. PSK is known to inhibit the protein expression of MMP2 and MMP-9 [63]. Luo et al. [62] results suggest that the effect of CV on MMP-9 occurs at the translational level. The CV aqueous extract tested may have contained too little PSK to have an inhibitory effect on MMP-2 expression [62] (Figure 4).

In vivo studies on an animal model (mice) showed that in individuals given an aqueous extract of CV (for 4 weeks at a dose of 1 g/kg by oral gavage, every day), the mass of tumors was smaller than in the control group and the number of tumor metastases to the lungs was significantly reduced. However, no obvious effect of CV on inhibiting liver metastasis was demonstrated. In the conducted study, no significant difference was found between body weight and activity of plasma enzymes such as AST (aspartate aminotransferase), ALT (alanine aminotransferase), ALP (alanine aminotransferase), and CK (creatine kinase) in the group of mice with 4T1 tumors and the control group, which indicates the lack or low toxicity of the aqueous extract of CV [62].

Late-stage breast cancer is characterized by bone metastasis and bone osteolysis [64]. Bone cells induce the expression of cytokines and chemokines, which promote the settlement of breast cancer cells in the bones and cause the influx of other primary cells of this tumor. Conservation of C-X-C motif chemokine receptor type 4 (CXCR4 and CXCL12), receptor activator of NF-kB (RANK), and RANK ligand (RANKL) has been shown to be of great importance for targeting breast cancer cells to bone [60].

**Figure 5:**
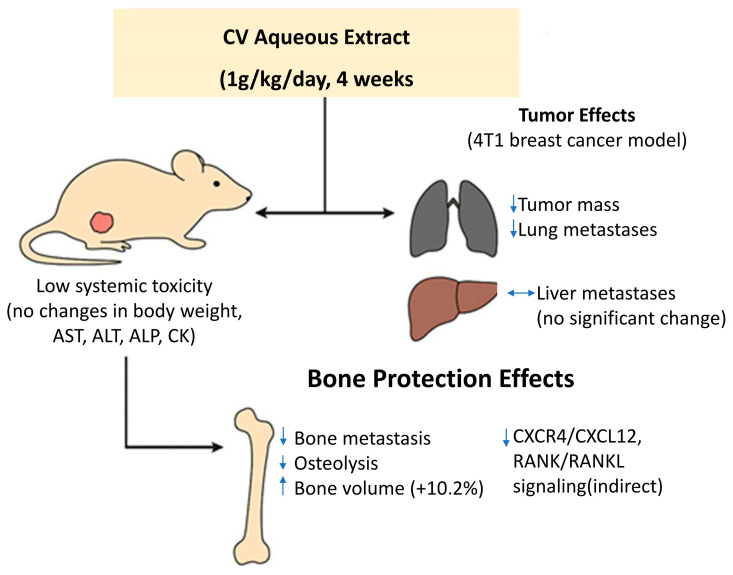
In vivo antimetastatic and anti-osteolytic effects of aqueous extract of *Coriolus versicolor* (CV) in a murine 4T1 breast cancer model. Daily oral administration of CV extract (1 g/kg for 4 weeks) significantly reduced primary tumor mass and lung metastases, without affecting liver metastasis. The treatment was well tolerated, with no changes in body weight or plasma enzyme activity (AST, ALT, ALP, CK), indicating low systemic toxicity. Additionally, CV extract exerted a protective effect on bone structure by reducing bone metastasis and osteolysis, increasing bone volume by 10.2%. These effects may result from indirect inhibition of CXCR4/CXCL12 and RANK/RANKL signaling pathways involved in bone colonization.

Administration of an aqueous extract of CV to mice with 4T1 tumors induced a protective effect on bone structure and an increase in its volume by 10.2%. The anti-osteolytic effect could result from the anti-tumor activity of the extract, reducing the metastasis of breast cancer cells to the bone, which consequently translates into the protection of the bone structure [62] (Figure 5).

In Yang’s studies [27], the anti-invasive properties of small molecules from *C. versicolor* (SMCV) were tested at concentrations of 25 and 50 μg/mL and 9-oxo-10E,12E-octadecadienoic acid methyl ester, the active compound AM, at a concentration of 25 μg/mL. The MDA-MB-231 breast cancer cell line, an adenocarcinoma with a high potential for metastasis, was used as a research model. It was shown that SMCV directly reduced the migration capacity of the breast cancer cells studied [27,65].

In conclusion, by suppressing MMP-9 expression, CV limits tumor cell invasion and migration, especially in triple-negative breast cancer models [62,66]. These findings provided scientific evidence for the possibility of clinical use of the aqueous extract of CV in patients with breast cancer [62,67,68].

### 3.5. Immunomodulation

Several works emphasize the role of CV extracts, especially protein-bound polysaccharide peptides (PBPs), in regulating the cell’s immune response. As previously established, *C. versicolor* fungus extract inhibits the secretion of proinflammatory cytokines by immune system cells in an inflammatory environment induced by LPS [24,69].

The modulatory effect of CV extract on the production of pro-tumour factors by MCF-7 breast cancer cells and human umbilical vein endothelial cells (HUVEC) in a pro-inflammatory microenvironment was investigated by Jędrzejewski et al. (2020) [66]. It is well known that inflammation increases the secretion of pro-inflammatory cytokines and metalloproteinases that promote tumour invasion and angiogenesis. Previous studies, including the findings of Jędrzejewski et al. (2016) [70] showed that CV extract can inhibit LPS-induced cytokine production in immune cells. In this study, MCF-7 cells in the presence of CV extract significantly decreased LPS-stimulated secretion of interleukin-6 (IL-6), a cytokine known to promote tumour proliferation, survival and metastasis. Additionally, a decrease in levels of IL-8, a chemokine associated with angiogenesis and tumour progression, was observed. The CV extract also suppressed MMP-9, a matrix metalloproteinase associated with poor cancer prognosis due to its role in tumour growth and spread. Interestingly, while endothelial cells treated with CV extract showed increased secretion of IL-6, IL-8 and MMP-9 compared to untreated cells, levels remained significantly lower than those induced by LPS. In addition, CV extract was found to mildly stimulate the production of several cytokines, including IL-1β, IL-2, IL-6, IL-10, TNF-α and IFN-γ, albeit to a much lesser extent than LPS. These findings suggest that CV extract shows the potential to inhibit the production of inflammation-related breast cancer-promoting cytokines [66,71,72]. Antimigratory properties and a cytotoxic effect of CV extract on MCF-7 cells were also stated, resulting from the increase in the amount of reactive oxygen species (ROS) [66].

In the experiment described by Jędrzejewski et al. (2020), a reduction in TLR4 (Toll-like receptor 4) expression and prevention of IκB p-phosphorylation were also observed in LPS-stimulated HUVEC and MCF-7 cells [66]. This effect is considered beneficial because in an inflammatory environment, the TLR4–NF-κB pathway is activated, which translates into inhibition of apoptosis, increased proliferation, migration and invasion of cancer cells [66,73,74,75].

CV extract has been shown to interact with immune cells via TLR4 receptors, and the degree of cytokine gene expression induced by LPS and CV is correlated with *NF-kB* gene transcription. The activation of the TLR4 signaling pathway by lipopolysaccharide (LPS) is a well-characterized mechanism that induces pro-inflammatory cytokine production. Under resting conditions, NF-κB p65 remains in the cytoplasm in an inactive state due to its association with the inhibitory protein IκB (inhibitor of nuclear factor kappa B). Upon LPS stimulation, a signaling cascade is initiated that leads to the phosphorylation and subsequent degradation of IκB, thereby releasing NF-κB p65. This active transcription factor translocates into the nucleus, where it promotes the expression of pro-inflammatory cytokine genes [76,77].

Kowalczewska et al. (2016) [78] conducted studies to assess the immunomodulatory and anticancer properties of polysaccharide peptides extracted from commercially available *C. versicolor* capsules (MycoMedica, Czech Republic) on an in vitro research model of MCF-7 breast cancer cells. Based on the obtained results, it was established that the tested PBPs at a concentration of 100 mg/mL inhibited the proliferation of MCF-7 cells after 48 h of exposure. Moreover, it was found that the level of IL-1b, IL-6 mRNA in MCF-7 cells after 18 h of incubation with PBP (100 and 300 µg/mL) from CV decreased compared to control cells. Interestingly, after 24 h, the level of *IL-6* mRNA expression increased by 6.93 ± 0.99 times higher than the control. A significant increase (by 41.48 ± 3.99 times) in the level of *TNF-α* (tumor necrosis factor α) mRNA expression in MCF-7 cells was noted after 24 h of PBP stimulation at a dose of 100 µg/mL. It has been established that TNF-α can activate p38 MAPK kinase and thus induce apoptosis in the cell. p38 MAPK (mitogen-activated protein kinase) kinase phosphorylates BCL-2 protein, blocking its antiapoptotic activity. As a consequence, this leads to the activation of caspase-9 and then 3 and 7 [79]. The authors found that the apoptotic effect in MCF-7 cells induced by polysaccharide peptides isolated from the CV fungus may be the result of direct action of PBP components on MCF-7 cells or indirect action through TNF-α receptors [78].

In Luo et al. [62] experiment, significant immunomodulatory effects were also obtained, resulting from the increased production of IL-2, IL-6, IL-12, TNF-α and IFN-γ in splenic lymphocytes of mice with 4T1 tumor treated with *C. versicolor* [62,67,68]. It is known that the immunostimulatory properties of CV, in particular its components such as PSK and PSP, activate T lymphocytes and increase the production of cytokines and antibodies. Previous results also showed that the ethanol–water extract of CV stimulates the Th1 lymphocyte response in vitro. The consistency of these observations with the current results confirms the strong immunomodulatory effect of CV, which may underlie its antitumor and antimetastatic properties [62,80].

Two publications by Jędrzejewski et al. from 2020 and 2024 [20,69] published very interesting results of studies on the effect of PBPs from the fungus *C. versicolor* on the interaction between 4T1 mouse breast cancer cells, which are the equivalent of stage IV TNBC breast cancer in humans, and macrophages (RAW264.7 line), the most numerous population of immune cells found in the tumor microenvironment, secreting factors involved in carcinogenesis.

The first study reported an experiment in which 4T1 breast cancer cells were cultured under several conditions: in conditioned medium (CM) derived from macrophages exposed to PBP (CM-PBP), in conditioned medium from macrophages not exposed to PBP, and in co-culture with macrophages to mimic the in vivo microenvironment.

The viability of 4T1 cells, the level of secreted cytokines and chemokines, the activity of arginase 1 and inducible nitric oxide synthase (iNOS) and the ability of cell migration were examined. The results of the studies showed that the medium after culturing macrophages without PBP stimulates the proliferation and migration of 4T1 cells. A higher concentration of proangiogenic factors secreted by cancer cells, VEGF and MCP-1 (vascular endothelial growth factor; monocyte chemoattractant protein-1), was also observed in these conditions.

However, culturing 4T1 cells in the medium after culturing macrophages with PBP caused inhibition of cell growth and migration, and decreased secretion of proangiogenic factors (VEGF, MCP-1). Moreover, examination of the level of markers for types M1 and M2 macrophages in the medium with the addition of PBP after simultaneous culture of cells of both lines showed a significant decrease in markers of the M2 subtype (arginase 1 activity, IL-10 and TGF-β—transforming growth factor β concentrations), and an increase in markers of the M1 subtype (iNOS activity and production of IL-6 and TNF-α). In the experiment, the authors confirmed that PBP changes the microenvironment favorable for tumor development and inhibits communication between 4T1 cancer cells and macrophages. PBPs with CV also affect the regulation of the production of angiogenic and inflammatory mediators. These results are of great importance because the treatment of patients with TNBC is limited due to the lack of identifiable, specific cell markers [69].

The results presented in the following publication refer to 4T1 triple-negative breast cancer (TNBC) cells treated with the CV fungal extract, which created a microenvironment that reduced macrophage viability and migration, as well as intracellular ROS formation.

**Figure 6:**
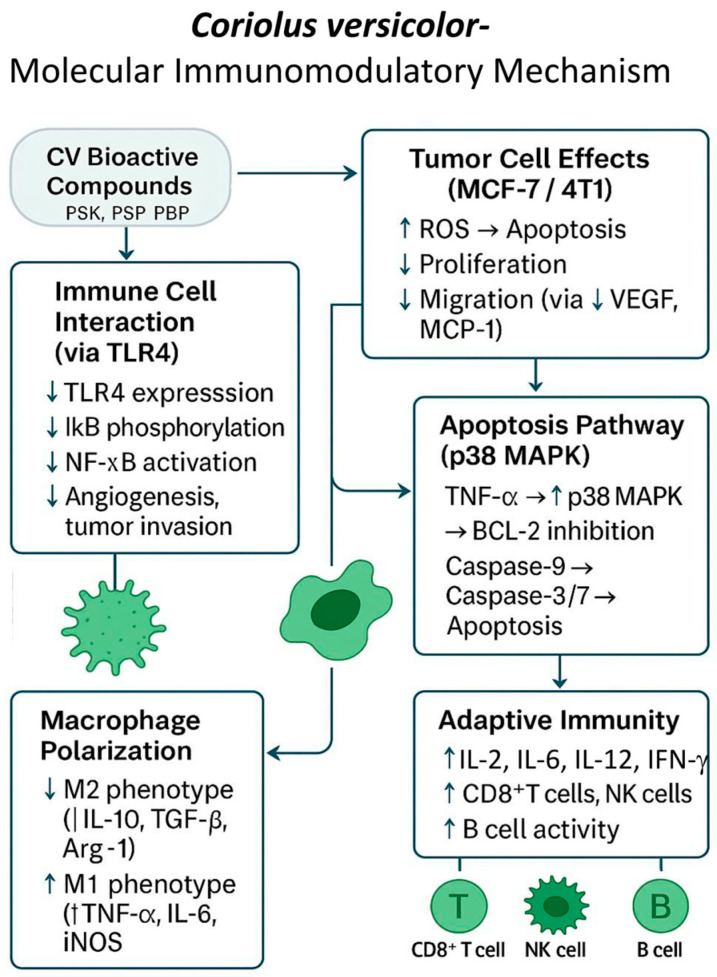
Molecular immunomodulatory mechanism of *Coriolus versicolor* (CV) bioactive compounds in the tumor microenvironment. CV-derived protein-bound polysaccharides (PSK, PSP, PBP) modulate immune responses by inhibiting TLR4/NF-κB signaling, reducing pro-inflammatory cytokines (IL-6, IL-8), and suppressing angiogenesis and metastasis via downregulation of VEGF, MCP-1, and MMP-9. CV promotes apoptosis in cancer cells through the p38 MAPK pathway and enhances M1 macrophage polarization (↑TNF-α, IL-6, iNOS), while inhibiting M2 phenotype markers (↓IL-10, TGF-β, Arg-1). Additionally, it stimulates adaptive immunity by increasing IL-2, IL-12, IFN-γ, and the activity of CD8^+^ T cells, B cells, and NK cells, collectively reshaping the tumor microenvironment toward antitumor immunity.

The level of synthesis of VEGF and MCP-1 cytokines stimulating angiogenesis also decreases. In this study, the method of culturing RAW264.7 macrophages in the medium after culturing 4T1 cells stimulated with *C. versicolor* extract was also used [20]. Thus, CV increases the production of TNF-α, IL-6, and other proinflammatory cytokines, promoting M1 polarization of tumor-associated macrophages (TAMs) and reducing angiogenic factors such as VEGF and MCP-1 [20,69] (Figure 6).

These combined actions position CV as a promising adjuvant that not only suppresses tumor growth but also remodels the tumor microenvironment to favor immune activation and reduced metastasis.

The analyzed publications also included a paper discussing the results of phase I clinical trials [21]. The aim of this study was to assess the safety, tolerability, and potential benefits of taking a preparation from *C. versicolor* by women with breast cancer after chemotherapy and radiotherapy. The study ultimately included 9 women with breast cancer who had completed radiotherapy. They were women aged 38 to 68 years diagnosed with stage I, II or III breast cancer, three with ER-positive and six with ER-negative cancer. The maximum tolerated dose of the CV preparation was initially assessed, and then the patients were divided into three groups, depending on the dose of powdered, lyophilized CV mycelium they received: 3, 6, or 9 g/day in two doses.

During the study, patients were monitored for adverse events (AE) and dose-limiting toxicity (DLT) criteria. Based on the study results, it was found that the CV preparation taken at a dose of up to 9 g/day was well tolerated. An increased number of lymphocytes was observed in women taking doses of 6 and 9 g/day, greater NK cell activity at the dose of 6 g/day, and a dose-dependent increase in the number of CD8^+^ T cells and CD19^+^ B cells. Therefore, it was concluded that the tested preparation of powdered CV mycelium may strengthen the immune status in patients with breast cancer and after standard oncological treatment [21] (Table 2).

**Table 2:** Selected in vitro and in vivo studies of CV’s influence on breast cancer.

No.	Reference	CV Formula	Research Model	Research Methods	Research Objective	Results/Effect Mechanism	Conclusions
1	Yuzugullu Karakus Y, et al. 2025 [49]	TPP separated laccase	MCF-7, MDA-MB-231	WST-1 ELISA analysis of Annexin V	assessment of the cytotoxic potential of laccase	laccase at high concentrations inhibited the viability of MCF-7 and MDA-MB-231 cells and caused apoptotic morphological changes in cells	laccase treatment was significantly more effective in MCF-7 cells than in MDA-MB-231 cells
2	Chauhan PS, et al. 2019 [54]	immobilized laccase from *C. versicolor* on Pluronic-stabilized silver nanoparticles	MCF-7	semi qRT-PCR experiments	to assess the anticancer efficacy of immobilized laccase in breast cancer MCF-7 cells	inhibition of MCF-7 cells proliferation through β-estradiol degradation and cell apoptosis	described new strategy increasing the activity of immobilized laccase provides new opportunities for its use in the development of hormone-dependent breast cancer therapy
3	Pawlikowska M, et al. 2020 [59]	CV capsules/PBPs	MCF-7	MTT HT-microscopy, DCF-DA, qRT-PCR, IF-based assay	to evaluate whether PBP extract triggers the necroptotic pathway in MCF-7 breast cancer cells	PBPs from CV induced RIPK1/RIPK3/MLKL-dependent necroptosis in MCF-7 cell line;	stimulating necroptosis has potential as an anticancer therapy
4	Kowalczewska M, et al. 2016 [78]	CV capsules, PBPs extract	MCF-7	MTT qRT-PCR;	to investigate the immunomodulatory and anticancer properties of the PBPs isolated from commercially available capsules of *C. versicolor*	the PBPs induced a significant decrease in breast cancer MCF-7 cells growth, in TNF-α-dependent manner; the level of IL-1β and IL-6 was not affected	further studies are needed to clarify the mechanism of PBPs in therapy and prevention of human cancers
5	Jędrzejewski T, Sobocińska J, et al. 2020 [66]	CV extract	MCF-7	MTT, LDH, ROS levels—DCFH-DA, wound-healing assay (Scratch Assay), cytokine and matrix metalloproteinase assays-ELISA, WB	to study the effect of CV extract on MCF-7 breast cancer cells in a pro-inflammatory microenvironment mimicked by lipopolysaccharide (LPS)	inhibition of the production of pro-inflammatory and pro-angiogenic factors by LPS-stimulated cells; reduction in the expression of TLR4 and p-IκB; anti-migratory and cytotoxic effect, an increase in ROS production	CV extract is cytotoxic to MCF-7 cancer cells and inhibits the expression of pro-tumor factors associated with inflammation
6	Shnyreva AV, et al. 2018 [33]	CV hot water extract; methanol: chloroform (1:1) extract	HBL-100, T-47D	MTT	to evaluate antiproliferative properties of CV extracts	the methanol–chloroform extract of *C. versicolor* (strain It-1) inhibited the growth of HBL-100 and T-47D cells	*C. versicolor* shows potential as a natural source for research and production of new chemical compounds with antiproliferative activity.
7	Yang CL, et al. 2023 [27]	CV extract 95% ethanol and small molecules from CV	MDA-MB-231	tumor cell invasion assay	to examine the anti-invasive effect of (SMCV)/bioactive compound	direct reduction in the invasive capacity of malignant MDA-MB-231 cells	SMCV bioactive compound has therapeutic potential, additional studies needed
8	Jędrzejewski T, et al. 2025 [20]	CV extract; the MycoMedica Company (Police nad Metují, Czech Republic).	4T1	MTT, ELISA (total protein, cytokine concentrations)	to assess whether triple-negative breast cancer (TNBC) cells incubated in the presence of CV extract can release factors that lead to a change in the macrophage subtype from pro-tumor to anti-tumor	TNBC cells stimulated with CV extract create a microenvironment that promotes reduction in macrophage viability and migration, intracellular ROS production and production of proangiogenic cytokines, and changes macrophage polarization toward an antitumor subtype	CV extract has the potential to be used in the treatment of TNBC-associated macrophages
9	Jędrzejewski T, et al. 2020 [69]	CV capsules, PBPs extract	4T1	MTT ELISA	study of communication between triple-negative mouse breast cancer 4T1 cells and macrophages in the presence of PBPs	PBPs inhibit communication between 4T1 cells and macrophages by regulating the production of angiogenic and inflammatory factors and promote the M1 macrophage phenotype in the neoplastic microenvironment	PBPs show chemopreventive potential and are promising agents for preventing the progression of TNBC
10	Luo KW, et al. 2014 [62]	CV aqueous extract (0.125–2 mg/mL); 1 g/kg, orally administered daily for 4 weeks	4T1, Female BALB/c mice (6–8 weeks of age) bearing orthotopic 4T1 tumors	MTT, scratch wound healing assay, transwell migration assay, gelatin zymography, WB, assessing the hematobiochemical markers in blood, H&E staining, micro-computed tomography (m-CT) analysis, ELISA	to examine the antitumor and antimetastatic properties of CV aqueous extract in 4T1 mouse breast cancer cells and in a 4T1 tumor-bearing mouse model	antitumor, antimetastatic and immunomodulatory effects in a mouse model of metastatic breast cancer; inhibition of the proliferation of 4T1 cells, cell migration and invasion; suppression of the activity of MMP-9 enzyme and protein level In vivo studies: effective reduction in tumor weight and lung metastasis; immunomodulatory effects	studies provided evidence supporting the use of CV aqueous extract as a health supplement in breast cancer patients
11	Torkelson CJ, et al. 2012 [21]	CV freeze-dried mycelial powder (Fungi Perfecti, Inc., Olympia, WA, USA) encapsulated by Beehive Botanicals (Hayward, WI, USA).	Phase I clinical trial; 9 women diagnosed with stage I, II, or III breast cancer	a phase I, two-center, dose escalation study; monitoring for: adverse events (AEs) using clinical and laboratory methods, dose-limiting toxicity (DLT) criteria defined as any Grade 2 or greater treatment-related toxicity as scored using the NCI’s Common Terminology Criteria for Adverse Events V 3.0 (CTCAE).	to assess the safety and tolerability of CV in women with breast cancer after radiotherapy; a collection of preliminary data comparing baseline values before and after radiotherapy, complete blood count with differential, immunological measurements during and after treatment, NK (natural killer) cell activity, regulatory T cell test complete, T/B/NK cell subset assay, phagocytic index, and cytokine level	CV was well tolerated. Immunological results indicated increased lymphocyte counts, increased NK cell functional activity, dose-dependent increases in CD8^+^ T cells and CD19^+^ B cells	CV therapy administered orally may improve the immune status of immunocompromised breast cancer patients undergoing standard oncological therapy

Abbreviations used in Table 2. 4T1—murine triple-negative breast cancer (TNBC) cells (Mimics human breast cancer stage IV); AEs—adverse events; CTCAE—Common Terminology Criteria for Adverse Events V 3.0; DCFH-DA—2′,7′-dichlorodihydrofluorescein diacetate staining; DLT—dose-limiting toxicity; ELISA—Enzyme Linked Immunosorbent Assay; HBL-100—human breast tumor cell line; HT—holotomographic microscopy; LDH—lactate dehydrogenase, cell in vitro viability assay; LPS—Lipopolysaccharides; MCF-7—human breast adenocarcinoma cell line with positive estrogen and progesterone receptors; MDA-MB-231—adenocarcinoma, highly aggressive, invasive and poorly differentiated triple-negative breast cancer cell line; MLKL—mixed lineage kinase domain-like protein; MTT—(3-(4,5-dimethylthiazol-2-yl)-2,5-diphenyltetrazolium bromide) cell in vitro viability assay; NCI—National Cancer Institute; NK—natural killer; PBPs—protein-bound polysaccharides; qRT-PCR—Quantitative Reverse Transcription Polymerase Chain Reaction; RIPK1—receptor-interacting protein1 kinase; RIPK3—receptor-interacting protein 3 kinase; ROS—Reactive Oxygen Species; TPP—Three-phase partitioning; WB—Western blot analysis.

The frequency of positive results in all 11 studies analyzed (100%) suggests a significant correlation between the use of CV extracts and reduced breast cancer cell viability or favorable modulation of the immune response.

The collected data indicate that *Coriolus versicolor* and its bioactive components, in particular PBPs and laccase, exhibit multidirectional antitumor activity in breast cancer models. These effects are particularly promising in the context of TNBC therapy.

## 4. Therapeutic Potential and Limitations of *Coriolus versicolor*

Although the number of high-quality clinical trials is limited, the results to date suggest that *C. versicolor* may improve quality of life and immune parameters in patients after chemotherapy, potentially increase the efficacy of adjuvant treatment, and be used as a safe adjuvant to conventional therapy [19].

A pilot, randomized, controlled clinical trial by Torkelson et al. (2012, USA) [21] that assessed the effect of taking 6 g daily of *C. versicolor* extract in women after chemotherapy for breast cancer confirms the high tolerability of CV-containing preparations [21]. However, larger, randomized clinical trials with adequate placebo control are needed to fully confirm the efficacy and safety of this fungus in the treatment of breast cancer.

An important step in this direction seems to be the identification of the mechanisms of action of active compounds of CV, a more precise molecular definition of their molecular targets in a transgenic mouse model, and a strategy for loss of function of probable targets. The composition and structure of the compounds also require detailed analyses, especially the peptide part of PSP, which would provide information on cell surface receptors. In vivo studies using purified components will enable confirmation of the results of in vitro experiments on the inhibition of breast cancer cells by fungal extracts. Currently, advanced analytical and medical techniques enable the development of more effective drugs based on the analysis of the structure and their mechanisms of action, which helps to improve the effectiveness of therapy and reduce adverse effects [24].

Studies on the induction of apoptosis in MCF-7 cells through increased activity of laccase immobilized on silver particles facilitate the understanding of the anticancer functioning of laccase and open up various options for its therapeutic use, especially in the treatment of hormone-dependent breast cancers [54]. Over the past several decades, drugs that induce an apoptotic effect in cancer cells have been designed and used in oncological treatment. The studies of Pawlikowska et al. (2020) [59] on the induction of necroptosis and the use of this phenomenon as anticancer therapy present promising results in the treatment of breast cancer. New information on the molecular mechanism of the action of PBPs on breast cancer cells in vitro, as the authors themselves emphasize, is of clinical importance, but requires validation in in vivo studies [59,81,82,83].

Given the growing body of data supporting the therapeutic effects of *C. versicolor*, further translational studies are needed to enable the practical use of preparations from this fungus in the clinical care of patients. Importantly, these analyses concern only women, which limits the possibility of generalizing the results to the male population with this cancer [84].

Patients often experience complications related to surgical treatment, chemotherapy, radiotherapy, and supportive therapies. Managing these symptoms, both physical and psychosocial, remains a current clinical challenge. It requires optimizing treatment strategies, including establishing adequate monitoring schedules and effective ways to prevent and treat both early and late adverse effects of oncological therapies [13].

**Figure 7:**
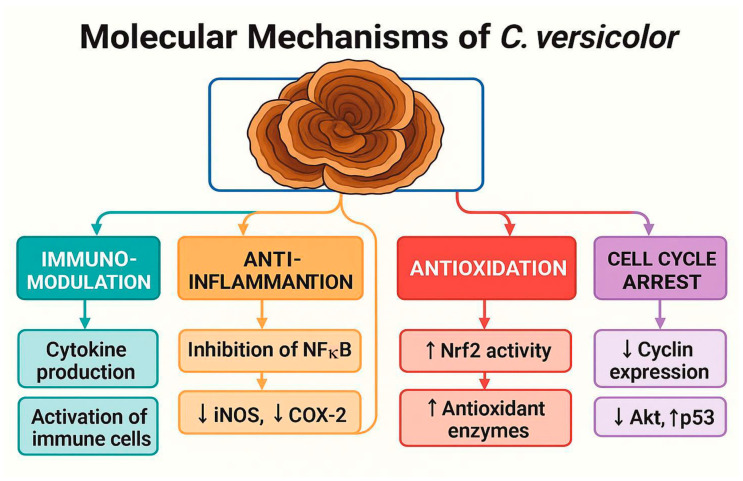
Molecular Mechanisms of *Coriolus versicolor* in Cancer Therapy. Schematic representation of the key biological effects of *C. versicolor* and its bioactive compounds. The mushroom exerts immunomodulatory effects by enhancing cytokine production and activating immune cells; anti-inflammatory effects through NF-κB inhibition and suppression of iNOS and COX-2; antioxidative effects by upregulating Nrf2 and antioxidant enzymes; and cell cycle arrest via downregulation of cyclins, Akt signaling, and upregulation of p53. These mechanisms contribute to its antitumor potential and therapeutic relevance in oncology.

Despite promising data, there are several significant limitations, such as the aforementioned lack of large clinical trials that could provide a basis for clinical recommendations or the heterogeneity of preparations and doses in the studies used, which makes it difficult to compare study results and implement therapeutic standards. There are also insufficient data on the pharmacokinetics and pharmacodynamics of PSK and PSP in humans and the possibility of interactions of substances from *C. versicolor* with anticancer drugs, although the data to date do not indicate significant clinical interactions. It is believed that the global incidence of breast cancer is increasing and this phenomenon will continue; therefore, it is necessary to unravel the multifaceted nature of this disease in order to develop effective therapeutic strategies [9] (Figure 7).

## 5. Limitations

In this review, articles published outside the PubMed database were excluded, which may result in missing important articles, but this should be considered a methodological limitation.

## 6. Conclusions and Perspectives

A growing body of evidence from in vitro and in vivo studies indicates that bioactive constituents of *Coriolus versicolor*, particularly polysaccharopeptide (PSP), polysaccharide-K (PSK), and protein-bound polysaccharides (PBPs), exert pronounced antiproliferative, pro-apoptotic, necroptotic, and immunomodulatory effects in breast cancer models. Among the tested preparations, PSP and PBPs appear especially promising for clinical translation owing to their reproducible activity across distinct breast cancer subtypes, their ability to reshape the tumor microenvironment, and their prior therapeutic use as adjuncts in other malignancies. Moreover, early-phase clinical findings with CV mycelial preparations have confirmed a favorable safety profile and good tolerability, further supporting their candidacy for integration into oncological care.

Several steps remain essential to advance *C. versicolor* towards clinical application in breast cancer therapy. Foremost, the development of standardized preparations with clearly defined bioactive components is required to ensure consistency across studies. Comprehensive pharmacokinetic and pharmacodynamic analyses in humans should follow, in order to establish optimal dosing regimens and assess potential interactions with conventional anticancer drugs. Early-phase clinical trials (phase I) must then evaluate safety, tolerability, and immunological responses in breast cancer patients, providing the basis for subsequent phase II studies focused on therapeutic efficacy, both as monotherapy and in combination with established modalities such as chemotherapy, radiotherapy, endocrine therapy, or immunotherapy. Ultimately, confirmatory evidence from large, randomized, placebo-controlled trials will be indispensable to support regulatory approval and routine clinical use.

In summary, *C. versicolor*—particularly preparations enriched in PSP and PBPs—should be regarded as among the most promising natural adjuvants for breast cancer management. Their immunomodulatory properties, favorable safety record, and encouraging preliminary clinical observations position them as realistic candidates for translational development, provided that the outlined research priorities are systematically pursued.

## Figures and Tables

**Table 1:** Chemical composition of *Coriolus versicolor* based on selected literature.

Compound Class	Specific Compounds/Examples	Biological Activities	References
Polysaccharides	Polysaccharopeptide (PSP), Polysaccharide-K (PSK)	Immunomodulatory, anti-tumor, TLR2 agonist, NK cell activation	[16,24,38,39,40]
β-Glucans	β-(1→3)-D-glucan, β-(1→6)-D-glucan	Immune stimulation, apoptosis induction	[3,39,42,43]
Proteins and Glycoproteins	Lectins, Fungal immunomodulatory proteins (FIPs)	Anticancer, immunostimulatory	[37,43,44]
Phenolic Compounds	Gallic acid, Catechin	Antioxidant, radical scavenging	[3,19,45]
Triterpenoids	Ergosterol, Lanostane-type triterpenes	Anticancer, anti-inflammatory	[3,19,43]
Sesquiterpenes	Spiroaxane derivatives, Rosenonolactone derivative	Potential antitumor properties	[46]
Sterols	Ergosterol	Cytotoxic, antioxidant	[3,19]
Enzymes	Laccase, Peroxidases	Biodegradation, antioxidant	[45]

## Data Availability

The original contributions presented in this study are included in the article. Further inquiries can be directed to the corresponding author(s).

## Abbreviations

The following abbreviations are used in this manuscript:

4T1	Murine triple-negative breast cancer (TNBC) cells (Mimics human breast cancer stage IV)
AEs	Adverse events
CTCAE	Common Terminology Criteria for Adverse Events V 3.0
CV	*Coriolus versicolor*
DCFH-DA	2′,7′-dichlorodihydrofluorescein diacetate staining
DLT	Dose-limiting toxicity
ELISA	Enzyme-Linked Immunosorbent Assay
HBL-100	Human breast tumor cell line
HT	Holotomographic microscopy
LDH	Lactate dehydrogenase, cell in vitro viability assay
LPS	Lipopolysaccharide
MCF-7	Human breast adenocarcinoma cell line with positive estrogen and progesterone receptors
MDA-MB-231	Adenocarcinoma, highly aggressive, invasive and poorly differentiated triple-negative breast cancer (TNBC) cell line
MLKL	Mixed lineage kinase domain-like protein
MTT	(3-(4,5-Dimethylthiazol-2-yl)-2,5-Diphenyltetrazolium Bromide) cell in vitro viability assay
NCI	National Cancer Institute
Nec-1	Specific inhibitor necrostatin-1
NK	Natural killer
PBPs	Protein-bound polysaccharides
qRT-PCR	Quantitative Reverse Transcription Polymerase Chain Reaction
RIPK1	Receptor-interacting protein1 kinase
RIPK3	Receptor-interacting protein 3 kinase
ROS	Reactive Oxygen Species
T47D	Ductal carcinoma, a cell line derived from the pleural effusion of infiltrating ductal breast cancer
TLR 4	Toll-like receptor
TPP	Three-phase partitioning
WB	Western blot analysis
WST-1	Water-soluble tetrazolium salt, in vitro viability and proliferation assay
